# Impact of long‐haul airline travel on athletic performance and recovery: A critical review of the literature

**DOI:** 10.1113/EP091831

**Published:** 2025-03-23

**Authors:** Petros G. Botonis, Argyris G. Toubekis, David W. Hill, Toby Mündel

**Affiliations:** ^1^ School of Physical Education and Sport Science National and Kapodistrian University of Athens Athens Greece; ^2^ Applied Physiology Laboratory University of North Texas Denton Texas USA; ^3^ Department of Kinesiology Brock University St Catharines Ontario Canada

**Keywords:** chronotype, circadian misalignment, jet lag, physical performance

## Abstract

Participation in many important sport events (e.g., World championships, Olympics) requires athletes to fly >4 h and to cross several time zones. This transmeridian travel results in a transient desynchronization of the body's circadian rhythms due to a disconnect between the timing of the endogenous circadian oscillator and the external stimuli, manifested as ‘jet lag’. While recent reviews highlight the importance of managing jet lag, the time required for resynchronization of the internal clock and dissipation of jet lag symptoms has not yet been summarized. Further, although the literature reports that rapid transmeridian travel is detrimental for athletes’ performance, empirical evidence from studies involving athletes is equivocal. Herein, we summarize the evidence that the variability in responses to transmeridian travel can be attributed to differences in (i) travel (real vs. simulated, westbound vs. eastbound, time zones crossed, during normal waking hours vs. normal sleep time), (ii) testing (assessment of performance vs. factors related to performance), and (iii) timing of the testing (destination time vs. ‘body time’), and we offer the possibility that differences in (iv) teams, (v) traits, and (vi) tournaments may also be implicated. We focus on (i) aerobic power/endurance, (ii) anaerobic power and capacity, (iii) strength, and (iv) mood state, sleep quantity and quality, and jet lag symptoms in this literature review, which is limited to athletes or physically active participants, travelling west or east crossing four or more time zones.

## Abstract (highlights)

- **What is the topic of this review?**
  A critical examination of the existing knowledge regarding the effects of rapid transmeridian travel on physical performance, the timeline required for restoration of performance, and dissipation of the symptoms of jet lag.
- **What advances does it highlight?**
  Contradictory results indicate it is not yet possible to conclude that there is any particular amount of time needed to recover from rapid transmeridian travel. Notable factors to consider in future include direction of travel and time zones crossed, the specific tests used and how they relate to characteristics pertinent to an athlete and their performance.

## 1 INTRODUCTION

The 2024 Summer Olympic Games were held in Paris, and many elite athletes from all over the world traveled long distances crossing multiple time zones to participate in such an important event. For instance, athletes from the Americas had to travel eastbound to Paris, crossing four to eight time zones, and athletes from the Pacific Rim had to travel westbound to Paris, crossing six to ten time zones. Rapid travel across multiple time zones is associated with a transient desynchronization of the body's circadian rhythms as there is a disconnect between the timing of the endogenous circadian oscillator (the body's internal ‘clock’) and the external stimuli (‘Zeitgeber’, most important among them being the light–dark cycle) that also influence the body's circadian rhythms. The desynchronization of rhythms is manifested as ‘jet lag’. The American Academy of Sleep Medicine (2005) defines jet lag as a syndrome involving insomnia or excessive daytime sleepiness following travel across at least two time zones. Other notable symptoms of jet lag include daytime fatigue, confusion, headaches, decreased vigor and attention, as well as bowel irregularities (Waterhouse et al, 1997; Waterhouse et al., 2007).

Although several reviews of the literature (e.g., Leatherwood & Dragoo, 2013; Rossiter et al., 2022) have reported that rapid transmeridian travel is detrimental for athletes’ performance, actual research findings from studies involving athletes are somewhat equivocal. For instance, while several studies involving athletes (Chapman et al., 2012; Fowler, Duffield, Morrow, et al., 2015; Hill et al., 1993; Lemmer et al., 2002; Reilly et al., 2001) have reported that several factors related to athletic performance deteriorate significantly after travel, others have reported no significant effect on performance‐related variables (Bullock et al., 2007; Thomson et al., 2013) or inconsistent outcomes in performance‐related variables (Fowler, Duffield & Vaile, 2015; Fowler, Knez, et al., 2017). To date, the impact of long‐haul transmeridian travel on exercise performance has not been synopsized. Moreover, it is relevant for sports scientists and practitioners to be aware of the possible adverse effects transmeridian traveling has on exercise performance.

Sport scientists and coaches typically adopt general recommendations regarding how long an individual must spend at the destination before the body adjusts to the light–dark cycle of the new destination – that is, how many days it takes to resynchronize the internal clock with the external Zeitgeber. The recommendation from the position statement of the European College of Sport Science (Reilly et al., 2007) is that it takes half a day per time zone crossed in westbound travel and 1 day per time zone crossed in eastbound travel to be fully aligned with the new light–dark cycle. Consistent with these general guidelines, several studies have shown that, irrespective of travel direction, the symptoms of jet lag gradually dissipate after arrival and essentially disappear after 5 days at the destination (e.g., Fowler, Knez, et al., 2017; Lemmer et al., 2002). Interestingly, however, two recent studies have reported divergent results: Fowler, Knez, et al. (2017) concluded that recovery might be faster than usually believed, whereas Biggins et al. (2022) reported that following a rapid transmeridian travel across seven time zones, jet lag persisted for up to 13 days. Additionally, the popularized travel advice is based on controlled laboratory experiments, which may have relatively poor translation to elite athletes in the field (Atkinson et al., 2014; Thomson et al., 2013). While recent reviews have highlighted the importance of managing jet lag (Janse van Rensburg et al., 2021, 2020), the time required for resynchronization of the internal clock and dissipation of jet lag symptoms has not yet been summarized. This is important for sports scientists and practitioners who have to be aware of the timeline required for jet lag dissipation, in order to design effective training and/or recovery plans after arrival at a new destination.

In this review, we offer the possibility that the variability in performance‐related exercise variables and jet lag responses to transmeridian travel that have been reported in the literature may be attributed in part to differences in participants and or procedures. We provide evidence from the literature on how differences in (i) travel, (ii) testing, and (iii) timing of the testing appear to impact responses to transmeridian travel. We can provide only our perspectives on why differences in (iv) teams, (v) traits, and (vi) tournaments might also influence responses.

- Travel. Arguably, an important aspect of travel research is whether the travel is real or simulated. Other aspects of travel that may influence the responses are that travel may be westbound or eastbound, it may be across 2 to 12 time zones, and it may be undertaken during normal waking hours or during normal sleep time. The effect of travel has been the focus of many studies.
- Testing. An important aspect of testing is whether it involves assessment of sport performance itself or evaluation of factors that are related to performance. Different factors that are related to sport performance (sleep quality, mood state, strength, aerobic power, anaerobic capacity, symptoms of jet lag) may be more susceptible. There have been a number of different dependent variables in studies of jet lag.
- Timing of the testing. As noted above, the timing of the departure may influence the measured effects of travel, if only because of the impact on sleep loss. But the time of day of the testing is also important. Many aspects of, and factors related to, performance display circadian rhythmicity, generally evidenced as ‘better’ scores in the afternoon. Consider eastbound travel across six time zones: if participants’ baseline testing is carried out at 15:00 (when they are at their best) and then post‐travel testing is carried out at the same ‘clock time’ at the destination, then the latter testing will be performed at 09:00 ‘body time’ (and typically after a fitful night's sleep). Is the reduced performance due to jet lag, sleep disruption, or circadian rhythmicity in the performance? The effect of timing of testing has been addressed in some jet lag studies, as it can be a confounding factor.
- Teams. We shall give our perspective on how different teams might respond differently to transmeridian travel.
- Traits. We shall give our perspective on how different personality traits might moderate the responses to rapid transmeridian travel.
- Tournaments. We shall give our perspective on how the purpose of travel – to a tournament or from a tournament – might impact the severity of jet lag symptoms.

Unfortunately, while the possible influences of differences in travel, testing and timing of the testing have been addressed in some jet lag studies, these factors have been either overlooked or poorly discussed in many others. For example, a recent systematic review of the literature described the impact of long‐haul travel on exercise performance, without, however, discussing the changes in endurance, anaerobic indices and strength separately (Rossiter et al., 2022). As noted above, there is no strong consensus regarding the timeline required for performance recovery following transmeridian travel.

The present narrative review aims to provide an overview of the existing knowledge regarding the effects of transmeridian travel on physical performance, as well as the timeline required for restoration of performance and dissipation of the symptoms of jet lag after travel. Most sports performance depends to one degree or another on (i) aerobic power/endurance, (ii) anaerobic power and capacity, (iii) strength, and (iv) sport‐specific skills. In addition, it is arguably correct to assume that all sport performance is influenced by certain (v) psychophysiological states. In this review, we have focused on (i) aerobic power, (ii) anaerobic power and capacity, (iii) strength, and (iv) mood state, sleep quantity and quality, and a collection of jet lag symptoms. We also discuss the potential factors that have been identified as possibly explaining differences between studies, including the role of (i) travel, (ii) testing, and (iii) timing of the testing. In addition, we provide our perspective on the possible influences of (iv) teams, (v) traits, and (vi) tournaments in the manifestation of jet lag symptoms. We focus on the physiological processes that potentially underline performance outcomes following long‐haul airline travel. Our literature search was limited to athletes or physically active participants, whose travel (or simulated travel) was west or east across four or more time zones.

## 2 PHYSIOLOGY OF JET LAG/CHRONOBIOLOGICAL CONSIDERATIONS

**FIGURE 1:**
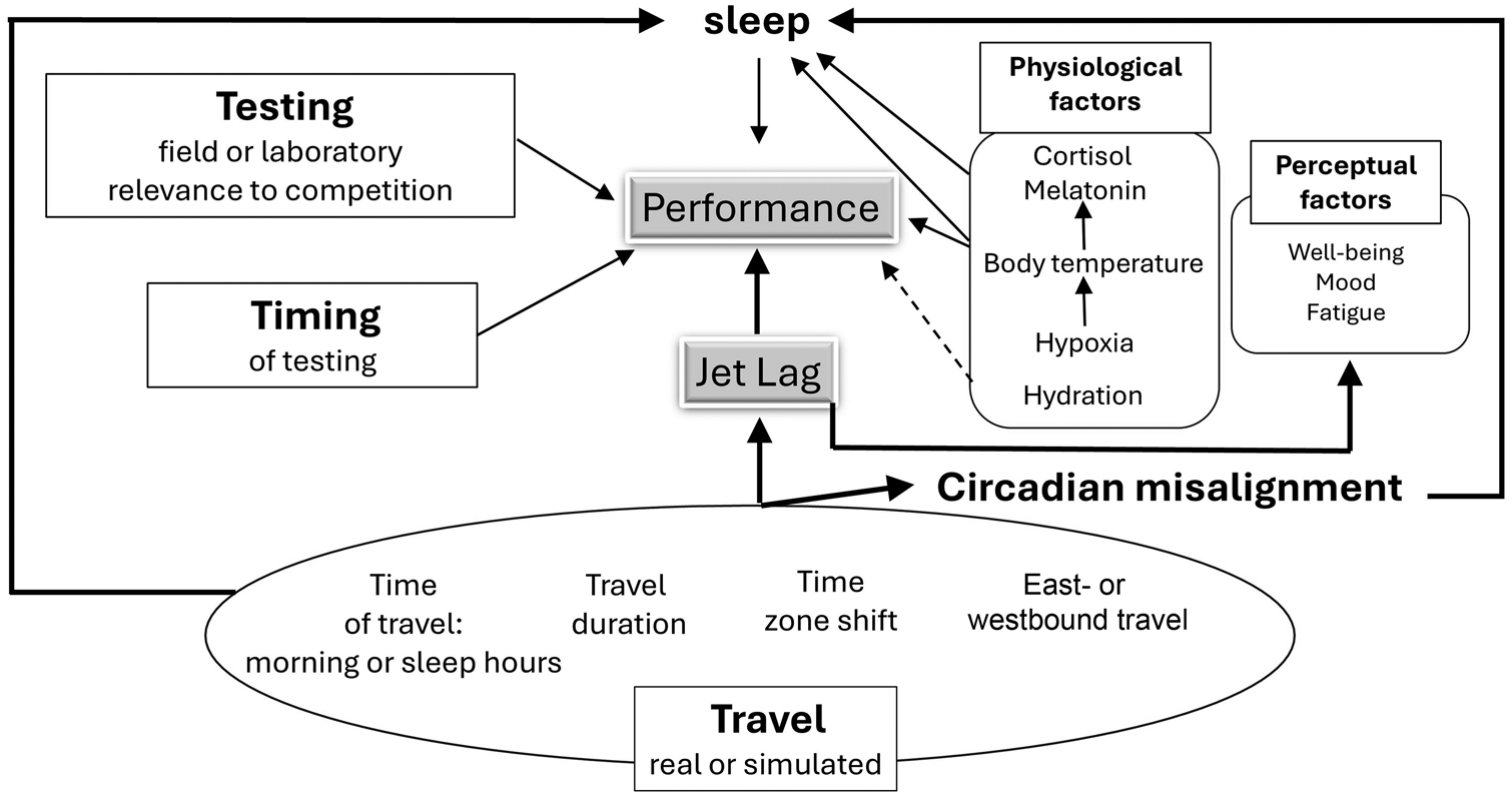
A schematic representation of underlying mechanisms affecting performance and jet lag responses following transmeridian travel.

Herein, we propose the main underlying physiological mechanisms that may affect jet lag following transmeridian travel. Traveling conditions and the physiological mechanisms combined with various variables that seem to be affected following long‐haul transmeridian travel are graphically represented in Figure 1.

### 2.1 Physiology of jet lag and circadian misalignment

Most of the physiological responses measured following travel demonstrate circadian rhythmicity. Several physiological processes (e.g., blood pressure, core body temperature, hormonal secretion and energy metabolism) present diurnal variation, that is, they demonstrate a maximum and a minimum value during the day. For instance, blood pressure decreases at night and increases toward the time of awakening (Douma & Gumz, 2018; Hower et al., 2018). Accordingly, core body temperature reaches its nadir at ∼04:00 to 05:00 in the morning and peaks late in the evening (Postolache et al., 2020; Vitale & Weydahl, 2017). Lemmer et al. (2002) were the first to investigate the influence of either a westbound or eastbound flight crossing six and eight time zones, respectively, on physiological responses measured at several time points within 11 days post‐travel. They noted that after travelling west, both systolic and diastolic pressure were elevated for 11 days after arrival and no differences were detected among the time points of testing. Moreover, the heart rate remained elevated in a similar manner. However, the opposite trend was observed for both systolic and diastolic pressure following the eastward travel, while no changes were observed for the heart rate profile. O'Connor et al. (1991) investigated the effects of either westward or eastward travel crossing four time zones on physiological responses of male and female collegiate swimmers. It was observed that females had lower and higher systolic pressure, compared to preflight values, after traveling west and east, respectively. By contrast, males exhibited unaltered blood pressure values. Regarding body core temperature, two studies noted that it resembled the normal early‐evening peaks and early‐morning nadirs (Lemmer et al., 2002; Reily et al., 2001) regardless of travel direction, but its trough time was lower on the first day after westbound travel (Lemmer et al., 2002).

With regards to hormones, melatonin (a mediator in the control of sleep/wake cycle) is synthesized in the darkness and, thus, it demonstrates maximum values at night (23:00 to 05:00), and cortisol reaches its peak and nadir early in the morning and late afternoon, respectively (Bellastella et al., 2019). Nonetheless, salivary cortisol of both sexes fluctuated similarly and, compared to preflight values, values were lower and higher after traveling west and east, respectively. Since the postflight saliva samples were obtained with 4 h advance or delay in relation to circadian phase at which preflight values were taken, it is speculated that the circadian phase of data collection might have affected the outcomes (O'Connor et al., 1991).

It should be recognized that there are factors inherent to the flight conditions that may play some role in jet lag and travel fatigue including the prolonged mild hypoxic exposure, the disruption of hydration/eating patterns and effects on sleep (daytime or nighttime flight). For example, in commercial air travel, cabins are ‘pressurized’ to provide a partial pressure of oxygen similar to that at an altitude of ∼5000 ft (1500 m). Thus, passengers experience prolonged mild hypoxic exposure, and a series of studies have shown that prolonged hypoxic exposure may partially explain changes in main circadian markers (i.e., core body temperature, melatonin and cortisol) as well as the sleep disturbances that are often observed after a transmeridian flight, independent of the number of time zones crossed (Coste, Beaumont, Batejat, Van Beers, Charbuy, et al., 2004, Coste, Beaumont, Batejat, Van Beers, Touitou, 2004, 2005; Coste et al., 2009). Coste, Beaumont, Batejat, Van Beers, Touitou (2004) for instance, showed a delayed evening fall in the body core temperature after an 8‐h exposure to mild hypoxia (12,000 ft/3700 m), thus indicating a phase delay. Likewise, both plasma cortisol and melatonin levels are affected by the hypoxic environment (Coste, Beaumont, Batejat, Van Beers, Charbuy, et al., 2004, 2005). In fact, an 8‐h exposure to hypoxia has been shown to induce a blunted cortisol response after its termination (Coste et al., 2005). In addition to effects on cortisol, an inhibitory effect on nocturnal melatonin secretion has been reported, even several hours after normoxia was re‐established (Coste, Beaumont, Batejat, Van Beers, Charbuy, et al., 2004). A subsequent study, employing the same hypoxic protocol, found a delayed occurrence of the core body temperature trough following hypoxia, which was accompanied by an increased sleep onset latency during the recovery night (Coste et al., 2009). These effects of hypoxia may explain, at least partially, the fatigue usually observed after long‐haul transmeridian flights, regardless of jet lag.

The disruption of hydration/eating status is also a factor that may contribute to travel fatigue, but it is still uncertain whether it affects the severity of jet lag symptoms. The dry air and the reduced oxygen partial pressure of the cabin result in increased water losses especially by evaporation of water at the skin surface (Greenleaf et al., 2004), and respiratory water loss, due to increased ventilation and increased need to humidify the air that enters the lungs (Waterhouse et al., 2007). So far, however, only a few studies have analysed the hydration status of athletes during long‐haul flights. Silva et al. (2016) showed that 51% of the kite surfer participants in their study self‐reported that they consumed less than 500 mL during a transmeridian flight, while the mean fluid loss rate is approximately 465 mL/h (Levkovsky et al., 2018). Nevertheless, the influence of hydration status and performance following transmeridian travel has yet to be examined in athletes (Janse van Rensburgh et al., 2020). With regard to eating patterns, Waterhouse et al. (2006) indicated that a limited selection of food availability – at least for those not traveling business class – as well as eating a meal at a ‘wrong’ biological time – at a time when biological clock indicates that passengers should be asleep – plays role in eating habit disruption. Nonetheless, an earlier study (Waterhouse et al., 2005) found that, although a simulated time‐zone transition to the east (eight time zones shift) was associated with eating disruptions, the degree of disruption had a weak relationship with the sensation of jet lag.

Transmeridian air travel desynchronizes biological rhythms, which, in turn, induces jet lag. It is well‐documented that the body's circadian (internal) clock is expressed in several, if not all, cells, and is coordinated by the superchiasmatic nucleus of the hypothalamus (Yoo et al., 2004). The temporary misalignment between the internal clock of the body, the destination time zone and the sleep/wake schedule is the main cause of jet lag following long‐haul travel, irrespective of direction. Τhe circadian misalignment between internal clock and external Zeitgeber (‘time‐givers’, including clock time) is an important mediator of sleep as it directly affects the variation of core body temperature. To date, several studies have shown that it is more difficult for the circadian clock to phase advance than to phase delay (e.g., Mitchell et al., 1997; Shanahan et al., 1999), and, therefore, the magnitude of jet lag symptoms appears to be greater after an eastbound than westbound flight because the resetting of the circadian clock takes longer following eastward than westward flight (Aschoff et al., 1975). This is partly attributed to the longer average free‐running period of humans’ circadian clock than 24 h (Czeisler et al., 1999). That is, a natural ‘day’ for the human body is ∼25 h, so it is easier to extend the natural day by 2 h after a westbound flight than to shorten it by 4 h after an eastbound flight.

### 2.2 Evidence of circadian misalignment

To date, 11 studies have documented the occurrence of jet lag symptoms following long‐haul airline travel, irrespective of destination (Table 1). The magnitude of jet lag, however, is dependent on the time zone shift. Concerning traveling with relatively short time zone transition, two studies that examined the effect of westward long‐haul airline travel crossing four and five time zones showed that jet lag symptoms do not persist for long (Fullagar, Duffield et al., 2016; Reilly et al., 2001). In particular, Reily et al. (2001) assessed the subjective rating of jet lag with a visual analogue scale and showed that jet lag was evident until 3 days following westbound airline travel with a five time zones shift, and it was more pronounced in the evening compared to the respective morning measures. However, jet lag measures returned to the predeparture values on the fifth day of arrival. Accordingly, Fullagar, Duffield et al. (2016) showed that traveling from the UK to South America (four time zones), largely increased the perception of jet lag (subjective rating of jet lag, sleep latency, sleep onset time, wake up time, inertia, fatigue, concentration, motivation, irritability, diet and bowel movements) on day 2 of arrival and remained moderately elevated up to the sixth day of arrival.

**TABLE 1:** Summarized findings of studies that investigated the effects of airline transmeridian flights on jet lag, psychophysiological variables and exercise performance parameters of athletes undertaken traveling to participate in a training camp or competition.

Reference	Participants	Experimental conditions	Departure and arrival time	Exogenous clock (local time of testing)	Endogenous clock (biological time of testing)	Physiological measures	Physical performance	Jet lag symptoms/perception
O'Connor et al. (1991)	22♂ and 18 ♀: college swimmers	(i) Westbound flight: Wisconcin‐Hawai, USA; four time zones (ii) Eastbound flight: Wisconcin‐Hawai, USA; four time zones	Westbound flight departure: 07:20, arrival: 15:45; Eastbound flight: departure: 17:20, arrival: 09:00	13:00–15:00	After westbound travel: 09:00–11:00 After eastbound travel: 17:00–19:00	Salivary cortisol: ↓ following westbound travel, ↑ following eastbound travel; systolic pressure: ↓ following westbound travel, ↑ eastbound travel (females), Ø (males)		RPE: Øparameters of profile of mood state were improved following travel regardless of destination
Hill et al. (1993)	7♀ footballers	Westbound flight: North America–Taiwan; eight time zones		Morning hours (unspecified time)	Afternoon hours (unspecified time)		Grip strength: ↓ 2 days following travel compared to baseline	Fatigue: ↑ 1 day following travel Vigour: ↓ 2 days following travel
Lagarde et al. (2001)	27 volunteers (19♂ and 8♀), fitness level: unspecified	Eastbound flight: Texas, USA–Landes, France; seven time zones	Departure time: 15:00 Arrival time: 06:00	Baseline tests: 2 days (09:00 and 14:00) and up to 10 days following arrival (09:00 and 14:00)	16:00, 21:00		Grip strength: ↓ (3.6–7.0% in the morning tests) Ø in the afternoon tests SJ and multiple jump tests: Ø	
Reilly et al. (2001)	8♂ athletes, 4♂ and 5♀ sedentary individuals	Westbound flight: UK–Florida, USA; five time zones Intervention: treatment (temazepam 10 mg, *n* = 9) vs. control group (*n* = 8)	Departure time: 14:00 Arrival time: 22:00	First day of arrival: 07:00, 12:00, 17:00, 21:00 h; days 3, 5, 7 at the same hours	02:00, 07:00, 12:00, 16:00	Tympanic temperature: Ø	Grip strength: ↓	Jet lag: ↑ Sleep quality: ↓
Lemmer et al. (2002)	19♂: athletes	(i) Westbound flight: Frankfurt–Atlanta; six time zones (*n* = 13) (ii) Eastbound flight: Munich–Osaka; eight time zones (*n* = 6)		Before departure (22:00, 23:30, 03:00, 07:00, 09:00, 10:30, 13:00, 16:00, and 20:00) and 1, 4, 6, and 11 days after arrival (morning and evening at the same hours)	After westbound travel: 04:00, 05:30, 09:00, 13:00, 15:00, 16:30, 19:00, 22:00 After eastbound travel: 04:00, 05:30, 09:00, 13:00, 15:00, 16:30, 19:00, 22:00, 02:00	Systolic pressure: ↑ Diastolic pressure: ↑ Heart Rate: ↑ Following westbound, opposite results after the eastbound flight	Grip strength: ↓a	
Bullock et al. (2007)	5♂ elite athletes that travelled vs. 7 elite athletes that did not travel	Eastbound flight: Canberra, Australia‐Calgary, Canada; 24 h flight; eight time zones	Departure time: 06:50 Arrival time: 13:50	Average time of cortisol measures: 06:45 Jet lag measures: 09:00, 13:30, 18:30 Average time of physical performance: 10:15	Average time of cortisol measures: 22:45 Jet lag: 01:00, 05:30, 10:30 Average time of physical performance: 02:15	Salivary cortisol: 67%↓ in the travel group	30‐m sprint: Ø	Jet lag: ↑ in 1, 2, 4, 7 d compared to baseline in travelled athletes
Dranitsin (2008)	12♂ and 1♀: junior rowers	Eastbound flight: Kiev, Ukraine–Beijing, China; five time zones shift		HRV measurements were performed before and after travel between 05:00 and 09:00	10:00–14:00	Ø Supine HRV day 1–3, day 8–10, day 12–17 ↓ Supine HRV day 8–12 ↓ Standing HRV day 4 and day 5		
Chapman et al. (2012)	National‐team skeleton athletes (*n* = 12); long‐haul travel group (4♀ and 1♂) vs. no travel athletes (6♀ and 1♂)	Eastbound flight: Canberra, Australia–Calgary, Canada; 24 h flight; eight time zones	Departure time: 06:50 Arrival time: 13:50	10:15	16:15		Travel group: Peak and mean SJ velocity: ↓ Peak and mean CMJ velocity: Ø CMJ height: ↓ SJ height: Ø	
Thomson et al. (2013)	20♀: elite footballers	Eastbound flight: New Jersey, USA–Lisbon, Portugal; 7 h flight; five time zones Intervention: light exposure (*n* = 11) vs. no light exposure (*n* = 9)	Departure time: 09:00 Arrival time: 04:00	Following arrival at: 08:00, 13:30, 18:00, 22:00	13:00, 18:30, 23:00, 03:00	Intra‐aural temperature: diurnal variation, ↑ in the light group on the first day postflight	Grip strength: diurnal variation, Ø between groups	Jet lag: ↑ at the first day of arrival and ↓ at the end of the 4‐day study in the light group
Fowler, Duffield, Morrow, et al. (2015)	13♂: physically active participants	24 h simulated westbound flight: Sydney, Australia–London, UK; eight time zone shift Intervention: bright light exposure + sleep hygiene vs. control		08:00 and 18:00 1 day before the flight and at 07:00 and 19:00 (AEST) (21:00 and 09:00; GMT) for 2 days post travel	Before the flight measures: 24:00 and 12:00; 23:00, 11:00	Sleep: ↓ Oxygen saturation: ↓ during travel HR in submaximal test: Ø	5‐ and 20‐m sprint: ↓ in the evening of day 2 and day 1 respectively Unloaded CMJ: ↓ in the evening YYIRT1: Ø	Vigor: ↓ Jet lag: ↑
Fowler, Duffield and Vaile, (2015)	10♂: physically active participants	24 h simulated westbound flight: Sydney, Australia–London, UK; eight time zone shift vs. control		16:00 1 day before the commencement of the travel, followed 24 h later by the travel at 09:00 and 16:00	01:00 and 08:00	Sleep: ↓ Oxygen saturation: ↓ HR submax: ↑ Cortisol: ↓	YYIRT1: ↓ CMJ: Ø Peak power or peak velocity: Ø	Fatigue: ↑ RPE: ↑ Physical feeling: ↓
Buchheit et al. (2016)	12♂: elite, professional football players	Eastbound flight: Paris, France–Hong Kong, China; 6 h time zone shift						Fatigue: ↑ 1 and 2 days after arrival RPE: ↑ on sixth day after arrival Sleep quality ↓ on the sixth day after arrival
Fullagar et al. (2016)	15♂ elite footballers	Westbound travel: 18 h; UK–South America; 4 h time zone shift	Departure time: 17:45	Perceptual and jet lag measures before travel and at 2, 4, 6, and 10 days following the arrival; Time of testing: unspecified	Unspecified			Jet lag: ↑ at the second day only Perception: Ø
Fowler, Knez, et al. (2017)	10♂: trained participants	(i) westbound flight: Sydney, Australia–Doha, Qatar; eight time zones); 4 days wash‐out (ii) Eastbound flight: Doha, Qatar–Sydney, Australia; eight time zones	Westbound departure time: 19:30, arrival time: 08:55 Eastbound departure time: 18:25, arrival time: 22:20	Two weeks before departure (09:00 and 17:00), and at the same time of day after westbound (during the first 4 days) and eastbound travel (during the first 4 days)	After westbound travel: 01:00 09:00 After eastbound travel: 17:00, 01:00	Sleep patterns (sleep onset and offset, sleep duration): ↓a	Following both westbound and eastbound flight: CMJ: ↓, 20‐m sprint test: ↓, a YYIRT1: Ø after westbound, ↓ after eastbound	Jet lag: ↑,a Motivation: ↓,a Physical feeling: ↓,a Fatigue: Ø after westbound
Fowler, McCall, et al. (2017)	22♂: elite footballers	19 h eastbound flight: Sydney, Australia–Vitoria, Brazil; 11 time zones	Departure: 12:25 Arrival: 20:30	Jet lag questionnaire: at 19:00 for 5 days after travel; wellness questionnaire: Before and following travel for 5 days at 09:00 local time	Jet lag questionnaire: 08:00 Wellness questionnaire: 22:00			Subjective sleep quality: ↓ up to 2 days post travel Sleep efficiency: ↓ in the day of arrival; large ES for difference between pre‐travel and 1–4 days post travel Mean wellness: ↓ the week following travel compared to pre‐travel Jet lag: ↑ 4 days post travel Training load: Ø
Fowler, Knez, Thornton, et al., (2021)	20♂: trained participants	21 h eastbound airline travel: Doha, Qatar–Perth, Australia; eight time zones	Departure: 18:25 Arrival: 22:20	09:00 and 17:00 on four days before travel and during four days after travel	17:00, 01:00	Sleep (time in bed, sleep onset, sleep duration, sleep efficiency): ↓	YYIRT1: Ø in the 1, 2, 4 days post travel	Jet lag: ↑ Function: ↓ Mood: Ø Motivation: ↓
Biggins et al. (2022)	20♂ and 21♀ elite football players	21 h eastbound airline travel: Ireland–Taiwan; seven time zones	Departure: 13:50 Arrival: 16:15	08:30 at baseline, 1–5 days (pre‐competition phase) and 6–18 days (competition phase) after arrival	15:30	Sleep (sleep duration and time in bed): ↓ 1–5 days after arrival		Jet lag: ↑ for up to 13 days after arrival; Sleep quality: ↓ 1–5 days and returned to baseline 6–18 days after arrival; Fatigue and Training load: ↓ at pre‐competition and competition phase compared to baseline Recovery, readiness to train, muscle soreness: Ø
Everett et al. (2022)	21♂: highly trained rowers	22 h westbound airline travel: Canberra, Australia–Milan, Italy; nine time zones	Departure: 16:00 Arrival: 14:00	17:00 approximately	08:00 approximately		CMJ height velocity: ↓ CMJ displacement velocity: ↓	

*Note*: The studies are presented in chronological order. ^a^Greater reduction following eastward flight. Abbreviations: ↑, increased following travel; ↓, reduced or deterioration following travel regardless of direction; AEST: Australian eastern standardized time; CMJ, countermovement jump; ES, effect size; GMT, Greenwich mean time; HRsubmax, submaximal heart rate; HRV, heart rate variability; Ø, statistically insignificant change; RESTQ‐sport, recovery stress questionnaire for athletes; RPE, rate of perceived exertion; SJ, squat jump; YYIRT, Yo‐Yo intermittent recovery test.

Concerning eastward travel, Thomson et al. (2013) randomized 22 world‐class female footballers to a bright light intervention or control group before the flight (from New Jersey, USA, to Lisbon, Portugal; five time zones). They observed that the subjective ratings of jet lag were greatest on postflight day 1 and decreased thereafter. Interestingly, the overall jet lag ratings (fatigue, motivation, hunger, meal satisfaction, sleep quality, bowel movement) were actually higher in the light group for approximately 24 h after the first light exposure, but these ratings decreased more substantially over the remaining postflight days, so that jet lag was rated as lower at the end of the 4‐day study in the group of light exposure compared with the control group.

When crossing more time zones, jet lag symptoms get even worse. So far, seven research studies (one of them employing simulated airline travel; Fowler, Duffield & Vaile, 2015) have shown that crossing from 6 to 14 time zones either west or east induces severe jet lag symptoms that persist for at least 4 days after arrival (Biggins et al., 2022; Bullock et al., 2007; Fowler, Knez, et al., 2017, Fowler, McCall, et al., 2017, Fowler, Knez, Thornton, et al., 2021; Lemmer et al., 2002). Following westward travel, two studies demonstrated that crossing six (Lemmer et al., 2002) and eight time zones (Fowler, Knez, et al., 2017) increased the perception of jet lag for 6 and 4 days post‐travel, respectively. As regards eastward travel, Lemmer et al. (2002) showed that elite athletes who crossed eight time zones reported themselves as jet‐lagged until the seventh day of arrival. Similar findings have been reported in three more recent studies (Fowler, Knez, et al., 2017, Fowler, McCall, et al., 2017, Fowler, Knez, Thornton, et al., 2021). Those studies reported that athletes who crossed eight time zones eastwards had increased jet lag ratings up to 4 days postflight (Fowler, Knez, et al., 2017, Fowler, Knez, Thornton, et al., 2021) and that an 11‐time zone airline travel induced significant jet lag until 5 days after arrival (Fowler, McCall, et al., 2017). Other studies have reported that eastbound airline travel across seven or eight time zones induced jet lag symptoms that persisted up to 10 (Bullock et al., 2007) and 13 days following arrival (Biggins et al., 2022), respectively. It has been purported that the magnitude and duration of jet lag symptoms are affected by the direction of the travel (Forbes‐Robertson et al., 2012). In line with this, the comparison of westbound versus eastbound travel has revealed that traveling east induces more severe and longer symptoms of jet lag compared to traveling west (Fowler, Knez, et al., 2017; Lemmer et al., 2002). A primary reason for the worse jet lag after an eastbound flight is the inadequate time for sleep. For instance, after traveling from the USA to Paris (e.g., eight time zones transition), sleep will be initially attempted at 14:00 (endogenous clock), and in that case, it will be difficult and fragmented. Regardless, the symptoms of jet lag dissipate, as the body's internal clock gradually resets to realign with the external Zeitgeber.

### 2.3 Circadian misalignment and perseverance of circadian rhythms in performance

Besides physiology, human performance presents circadian rhythmicity too. Anaerobic indices (e.g., sprint times, peak and mean anaerobic power in a Wingate test) and strength, peak between ∼13:00 and 21:00 (Drust et al., 2005; Knaier et al., 2022). We have reported that ${\dot{V}}_{O_{2}\max}$ is higher later in the day (Hill, 1996, 2014; Hill et al., 1994, 1992), and in addition ${\dot{V}}_{O_{2}}$ kinetics is faster (Hill, 1996, 2014; Hill et al., 1994) and anaerobic capacity is higher, whether it be quantified by work in a 30‐s Wingate test (Hill & Smith, 1991; Hill et al., 1992) or the gold standard maximal accumulated oxygen deficit (Hill, 1996, 2014; Marth et al., 1998). Nevertheless, the evidence suggesting circadian rhythmicity of endurance performance is considered to be weak (Knaier et al., 2022).

To date, five studies have demonstrated that circadian misalignment is likely a major factor underlying the confounding results. In fact, irrespective of exogenous clock, lower performance has been observed, when the endogenous clock corresponds to early morning hours, and core temperature usually reaches its nadir (Lagarde et al., 2001; Chapman et al., 2012; Fowler, Duffield, Morrow, et al., 2015; Fowler, Duffield & Vaile, 2015, Fowler, Knez, Thornton, et al., 2021). For instance, reduced (4–7%) and unchanged grip strength has been found in tests applied at 09:00 and 14:00 local hours (endogenous clock: 02:00 and 07:00, respectively; Lagarde et al., 2001). Furthermore, reduced jumping ability has been shown in tests applied in the morning (i.e., 09:30 to 11:00) and the endogenous clock corresponded to approximately 01:30 (Chapman et al., 2012). In addition, diminished sprinting times and intermittent endurance performance were shown in measures obtained in the evening (endogenous clock: 08:00) but not in the morning (endogenous clock: 22:00) (Fowler, Duffield, Morrow, et al., 2015). Notwithstanding, the above‐mentioned findings do not agree with other studies. For example, Fowler, Knez, et al. (2017) noted similar anaerobic and endurance performance in tests applied at 09:00 and 17:00 local hours (endogenous clock: 17:00 and 01:00, respectively). Likewise, a previous study (Thomson et al., 2013) showed that in tests applied across several time points within a day (08:00, 13:30, 18:00 and 22:00, local hours) handgrip strength remained unaltered. Collectively, a number of studies, but not all, suggest that circadian misalignment and time‐of‐day‐time are important parameters to be considered in data interpretation following long‐haul travel.

### 2.4 Circadian misalignment and sleep disturbances

Due to its physiological and psychological recuperative effects, sleep is an integral part of recovery for human beings, and athletes particularly (Halson, 2019). A typical sleep cycle is composed of periods of 90‐min cycles divided into periods of non‐rapid eye movement and rapid eye movement sleep (Shapiro et al., 1981). It is well‐documented that elite athletes may present inadequate habitual nocturnal sleep patterns (e.g., Koutouvakis et al., 2024) and that sleep during preparation for the Olympics might be significantly disturbed (Botonis & Toubekis, 2023).

Traveling across time zones disrupts the sleep–wake cycle contributing to the severity of jet lag symptoms and the decrement of exercise performance post‐travel (e.g., Biggins et al., 2022; Fowler, Duffield & Vaile, 2015). To date, six studies have objectively measured sleep before, during and after travel (Biggins et al., 2022; Fowler, Duffield, Morrow, et al., 2015, Fowler, Duffield & Vaile, 2015, Fowler, Knez, et al., 2017; Fowler, Knez, Thornton, et al., 2021; Fullagar, Duffield et al., 2016). The existing findings suggest that sleep disruption is not always the case following westbound travel, as sleep patterns may not be significantly altered after arrival at the new destination. To wit, Fullagar, Duffield et al. (2016) reported truncated sleep duration during flight and a ‘rebound’ effect (increase in sleep duration and sleep efficiency) post‐travel; while others have shown that even though sleep quantity was disturbed the day of the flight (∼5 h less), it returned to baseline levels after travel (Fowler, Duffield, Morrow, et al., 2015, Fowler, Duffield & Vaile, 2015). However, sleep disturbances are consistently observed following eastward travel (Biggins et al., 2022; Fowler, Knez, et al., 2017, Fowler, Knez, Thornton, et al., 2021). In particular, traveling east crossing seven or eight time zones reduces the average nocturnal sleep duration post‐travel by 27–64 min (Biggins et al., 2022; Fowler, Knez, et al., 2017, Fowler, Knez, Thornton, et al., 2021), delays sleep onset by approximately 77 min (Fowler, Knez, et al., 2017), and increases the average wake time (sleep quality indicator) by 24 min (Fowler, Knez, Thornton, et al., 2021) for 4–5 days post‐travel.

Sleep loss/disruption during westbound flights has been proposed as a main reason explaining performance deterioration post‐travel. In a study using simulated travel, Fowler, Duffield & Vaile, (2015) found significant sleep loss (∼4.5 h) the day of the travel and concomitantly reported suppressed performance in the Yo‐Yo intermittent recovery test (YYIRT). They also found an increased submaximal heart rate during exercise, as well as reduced cortisol secretion and worse physical feeling the day after. These are in line with previous indications that wakefulness induces psychophysiological stress, increases cardiac sympathetic activation, and decreases physical and mental activity (Meerlo et al., 2008).

Sleep disturbances after traveling east are often followed by significant exercise performance deterioration and increased jet lag symptoms, which are less pronounced after traveling west. Indeed, Fowler, Knez, et al. (2017) found that the significant reduction in sleep duration 2 days after travel was accompanied by less distance covered in YYIRT within the same days. In the following days (days 3 and 4), however, intermittent sprint performance reached the predeparture values, which presumably could be attributed to the progressive restoration of sleep. The importance of obtaining adequate sleep when traveling either west or east has also been highlighted by others (Fowler, Duffield, Morrow, et al., 2015; Fowler, Knez, et al., 2017, Fowler, Knez, Thornton, et al., 2021). The first study (Fowler, Duffield, Morrow, et al., 2015) proposed that reduced sleep duration after a simulated airline flight led to deterioration of maximal sprint and vertical jump, but not YYIRT, performance. Likewise, Fowler, Knez, et al. (2017) observed that physically trained men, who maintained their sleep quantity during and following travel crossing eight time zones west, presented unaltered intermittent sprint performance as well as unchanged 5‐ and 20‐m sprint time post‐travel. In addition, it is of interest to note that two studies (Fowler, Duffield, Morrow, et al., 2015, Fowler, Knez, Thornton, et al., 2021) employed sleep hygiene combined with light exposure protocols to improve sleep, jet lag symptoms and performance the days after travel. Although both studies showed that the protocols were efficient in improving sleep duration, they showed controversial results on performance. Fowler, Duffield, Morrow, et al. (2015) reported no improvements in maximal sprint, vertical jump and YYIRT performance; while more recently, leg power was enhanced post‐travel following such a protocol, but performance in 5‐m sprint time and YYIRT remained unaltered (Fowler, Knez, Thornton, et al., 2021).

Travel‐related sleep issues may occur because of the time of the flight (traveling at night vs. daytime traveling), waking up earlier for an early‐morning flight, sleep interruptions during flight as well as due to later bedtime and earlier waking‐up time after traveling east and west, respectively (Reilly et al., 2009). Sleep restriction (Fullagar, Skorski, et al., 2016; Sargent et al., 2014) or sleep disturbances can lead athletes to significant performance impairments (Craven et al., 2022; Fullagar et al., 2015). It is noteworthy that Craven et al. (2022) showed that the longer an athlete remains awake during the night, the greater the next‐day performance reduction will be. Indeed, important physical (endurance performance, anaerobic capacity and strength) and cognitive aspects of performance have been shown to decrease following sleep loss (Abedelmalek et al., 2013; Hurdiel et al., 2014; Souissi et al., 2013). Several physiological mechanisms have been proposed in the literature to explain performance deterioration after sleep loss. First, a night of sleep loss lessens the time spent in non‐REM sleep, and results in shortened time spent in slow‐wave sleep. This, in turn, may reduce the secretion of growth hormone, thus leading to diminished recovery via impaired muscle repair (Léger et al., 2018). Second, reduced basal and postexercise antioxidant capacity (Romdhani et al., 2019), as well as increased muscle damage biomarkers during (Mejri et al., 2017) and post‐exercise (Rae et al., 2017), has been observed in athletes following a night of partial sleep deprivation. Third, sleep loss may affect both the next‐day well‐being and sensation of effort (Cullen et al., 2020). Conversely, adequate and non‐fragmented sleep has been shown to increase subjective well‐being (participants feel better) and reduce the sensation of effort in athletes participating in different sports (Juliff et al., 2018; Mah et al., 2011). The mechanism behind the deterioration of well‐being and increased sensation of effort following sleep loss is still elusive. Nonetheless, evidence suggests that this might be linked to alterations in cytokines and neuroendocrine signalling factors (e.g., cortisol, adrenaline, noradrenaline and brain‐derived neurotropic factor) following sleep deprivation (Cullen et al., 2020).

Altogether, maintaining adequate sleep appears to play an essential role in the recovery process of the athletes following transmeridian flights regardless of direction. It is noteworthy that traveling east may disturb athletes’ sleep more than traveling west. The attenuation of sleep disruption through efficient sleep hygiene strategies may minimize the deleterious jet lag symptoms and facilitate the timely restoration of athletic performance.

## 3 EFFECTS OF TRANSMERIDIAN TRAVEL ON AEROBIC POWER/ENDURANCE

The ability to maintain high levels of aerobic power is paramount for endurance athletes (distance running, cycling), mixed demand events (requiring both aerobic and anaerobic metabolism contribution: middle distance running, most speed skating and cycling and swimming events, rowing) as well as for many team‐sports players. In the laboratory, maximal power is usually identified by maximal oxygen consumption (${\dot{V}}_{O_{2}\max}$), and sustainable power is usually identified by the ventilatory threshold, lactate threshold or critical power.

In the field, aerobic power is often estimated using shuttle run tests, which may be continuous or intermittent in nature. One such test is the YYIRT (Bangsbo et al., 2008). Test duration in the YYIRT is ∼5–20 min, suggesting that it falls in the severe exercise intensity domain and is capable of eliciting ${\dot{V}}_{O_{2}\max}$ or, at least, providing some indication of the value of the participant's ${\dot{V}}_{O_{2}\max}$ (Bangsbo et al., 2008).

To date, four studies have investigated the effect of long‐haul airline travel on measures of, or factors related to, aerobic power and/or endurance performance. All four studies used the YYIRT as the dependent measure (Fowler, Duffield, Morrow, et al., 2015, Fowler, Duffield & Vaile, 2015, Fowler, Knez, et al., 2017; Fowler, Knez, Thornton, et al., 2021). Two of the studies employed simulated air travel (Fowler, Duffield, Morrow, et al., 2015, Fowler, Duffield & Vaile, 2015). In both studies, participants were exposed to normobaric hypoxic rooms, where altitude corresponded to 2093 m (fraction of inspired oxygen: 17%) and environmental temperature was set at approximately 21°C.

The results of these studies are summarized in Table 1. In response to westward travel crossing eight time zones, performance on the YYIRT was shown to be reduced in one study (Fowler, Duffield & Vaile, 2015), but not in two others (Fowler, Duffield, Morrow, et al., 2015; Fowler, Knez, et al., 2017). Importantly, also, the studies by Fowler, Duffield, Morrow, et al. (2015) and Fowler, Duffield & Vaile, (2015) showed that physiological responses of submaximal exercise follow the same trend as performance. Indeed, along with deterioration of performance in YYIRT observed by Fowler, Duffield & Vaile, (2015) in the evening of the day after the travel, heart rate during submaximal exercise (5 min test) was significantly increased. Accordingly, submaximal exercise responses were unchanged when performance in the YYIRT was unchanged (Fowler, Duffield, Morrow, et al., 2015). Equivocal results are observed from the studies investigating endurance performance following eastward travel. Fowler, Knez, et al. (2017) reported that compared to preflight values, traveling from Doha, Qatar to Sydney, Australia (eight time zones shift) reduced performance in YYIRT in the first and second day, while it was restored in the third and fourth day, postflight. Conversely, unaltered performance in YYIRT has been demonstrated in the first 4 days after eastbound travel (eight time zones shift; Fowler, Knez, Thornton, et al., 2021).

With respect to our three potential factors to explain the effects of transmeridian travel, for (i) travel, two of the studies investigated the effects of an actual flight, either eastbound travel across eight time zones (Fowler, Knez, Thornton, et al., 2021) or both eastbound across eight times zones and then 10 days later, on the return to home, westbound across eight time zones (Fowler, Knez, et al., 2017); the other two employed simulated travel (exposure to normobaric hypoxic rooms). For (ii) testing, while the YYIRT is a well‐established and easy to administer test, it does not generate a value for any standard physiological parameter and is not the best indicator of endurance ability. For (iii) timing of the testing, in three of the studies (Fowler, Duffield, Morrow, et al., 2015, Fowler, Duffield & Vaile, 2015; Fowler, Knez, et al., 2017) testing at the simulated or real destination was carried out in the morning and in the evening to address whether performance was affected by circadian rhythmicity and higher performance was observed in the evening trials suggesting that circadian rhythmicity may have confounded the quantification of the effects of jet lag per se. We should also consider that we are not currently aware of the influence of transmeridian travel on endurance performance in constant‐power activities or how chronotype of the participants may have affected the findings, since only one study has examined the effect of chronotype on endurance performance following transmeridian travel indicating no clear effect.

Collectively, although studies involving the effect of rapid transmeridian travel on factors related to endurance performance have presented inconsistent results, they do seem to indicate that intermittent endurance performance is affected by an actual westbound flight, although the extent of the decrement is still uncertain; responses to eastbound travel, whether real or simulated, are somewhat equivocal. Regardless, whenever performance is reduced, recovery might be expected within 3 days after travel. Since only intermittent endurance performance has been tested, we postulate that the generalization of the results is likely limited to intermittent activities, which characterize most team sports. It might not be appropriate to generalize to constant‐power activities, like athletics, rowing, cycling or swimming.

## 4 EFFECTS OF TRANSMERIDIAN TRAVEL ON ANAEROBIC POWER AND CAPACITY

A large spectrum of sport events is dependent on anaerobic energy production. In very short duration, extreme intensity events (e.g., sprints) or in sports characterized by intermittent bursts of extreme intensity exercise (e.g., volleyball, water polo) (e.g., Botonis et al., 2019; Kalinski et al., 2002), the rate at which energy can be provided is crucial; this is quantified as the peak anaerobic power. In longer duration, extreme and severe intensity events requiring both aerobic and anaerobic metabolism contributions (e.g., middle distance running, most speed skating, cycling and swimming events, rowing, as well as for many team sports like football (soccer), the amount of energy that can be provided by the anaerobic pathways is crucial (e.g., Kalinski et al., 2002; Peyrebrune et al., 2014); this is quantified as anaerobic capacity.

Two studies of transmeridian travel (presented together in Hill et al., 1993) have assessed anaerobic power as peak 5‐s power in the 30‐s Wingate test, determined on the first 4 days at the destination. Fowler, Knez, Thornton, et al., (2021) and Fowler, Duffield & Vaile, (2015) used performance in 5‐m and 20‐m sprints to assess anaerobic power on the first 2 days at the destination after simulated or actual transmeridional travel. Bullock et al. (2007) showed that skeleton athletes, who travelled from Australia to Canada, demonstrated unchanged maximal 30‐m sprint performance throughout 10 days post‐travel. Anaerobic capacity was assessed as total work performed during the 30‐s Wingate tests in two of the three studies reported in Hill et al. (1993).

The results of these studies are summarized in Table 1. With respect to anaerobic power, Hill et al. (1993) reported 7% reductions in 5‐s peak power in a Wingate test on day 1 after westbound travel across five times zones by five women and four men, and non‐significant reductions of 10% on day 1, and 14% on day 2 after eastbound travel across five time zones by nine woman and one man. Two more recent studies reported reductions in 5‐m and 20‐m sprint performances on the first 2 days at the destination after simulated (Fowler, Duffield & Vaile, 2015) or actual (Fowler, Knez, Thornton, et al., 2021) transmeridional travel. With respect to anaerobic capacity, Bullock et al. (2007) showed that skeleton athletes, who travelled from Australia to Canada, demonstrated unchanged maximal 30‐m sprint performance throughout 10 days post‐travel.

With respect to our three potential factors to explain the effects of transmeridian travel, potentially confounding effects may be associated with them. For (i) differences in travel, for example, the eastbound travel in the Hill et al. (1993) occurred during normal sleeping hours and was associated with anecdotal reports of significant sleep disruption, whereas the westbound travel was during the participants’ normal waking hours and there were very few complaints of sleep disruption; also, three studies involved real travel and, one involved simulated travel. For (ii) testing, sprint tests are simple to administer after travel, but may not be the best indicator of anaerobic power, and Wingate testing has similar issues, not to mention that the exercise mode, cycling, is not the mode of most sports. For (iii) timing of the testing, for example, the Fowler, Duffield & Vaile, (2015) study clearly demonstrated the confounding interaction between circadian rhythms in performance and performance at the destination.

Collectively, the results of these studies suggest that anaerobic capacity (as quantified by work in a 30‐s Wingate test) and anaerobic power (as quantified by 5‐s power in a Wingate test or performance of a short sprint) are impacted after transmeridian travel, more so following eastbound travel. Performance deterioration may be expected to last up to 3 and 4 days after westbound and eastbound travel, respectively. For the explanation of the above mentioned results, we should consider that (i) Wingate cycling and running sprints are more pertinent to athletes involved in cycling and running than to swimming or rowing, and (ii) sex differences and chronotype were not examined.

## 5 EFFECTS OF TRANSMERIDIAN TRAVEL ON MUSCLE STRENGTH AND POWER

Many sports events require high levels of muscular strength and power. Each of these characteristics is expressed in a short duration activity that may be performed slowly (strength, such as performing a one‐repetition maximum in the squat exercise) or fast (power, such as performing a vertical jump). Any sport that involves jumping (e.g., volleyball, basketball, handball), throwing (e.g., shot put, water polo), or hitting (e.g., baseball, tennis) depends on muscle power. Many combat sports and other contact sports also require pure strength.

In the laboratory or weight room, muscle strength is usually determined as the one‐repetition maximum in a free‐weights exercise that is pertinent to sport performance. Many times, muscle strength is determined using a handgrip dynamometer, a low‐cost tool that measures force production in an isometric handgrip exercise; while grip strength itself has limited applicability (e.g., wrestling), it is often assumed that it is representative of overall body strength or that the effects on grip strength mimic the effects on more pertinent measures of strength. Muscle power is best assessed using an expensive computerized system that controls speed or angular velocity. Alternately, force platforms or technology that measures the speed at which force is applied or resistance is moved can be used to generate measures of peak force. A low‐cost estimate of peak power is the vertical jump. Although power is not measured, the amount of work performed (height × body weight) in as short a time as possible is indicative of peak power.

Compared to aerobic parameters, anaerobic performance measures are more feasible and more easily tested following a transmeridian travel. For this reason, probably, indexes of anaerobic power have been tested more than aerobic ones. Indeed, muscle strength and/or power has been evaluated in 13 different jet lag studies, eleven involving real travel (Bullock et al., 2007; Chapman et al., 2012; Everett et al., 2022; Fowler, Knez, et al., 2017, Fowler, Knez, Thornton, et al., 2021; Hill et al., 1993; Lagarde et al., 2001; Lemmer et al., 2002; Reily et al., 2001; Thomson et al., 2013; Wright et al., 1983) and two using simulated flights (Fowler, Duffield, Morrow, et al., 2015; Fowler, Duffield & Vaile, 2015).

### 5.1 Isometric strength

Isometric strength (i.e., grip strength) has repeatedly been shown to be affected during the first 1–3 days at the destination following westbound travel across 5–11 time zones (Hill et al., 1993; Lemmer et al., 2002; Reilly et al., 2001). However, inconsistent results were shown by two other studies investigating the influence of eastbound travel on static strength (Hill et al., 1993; Lagarde et al., 2001). For instance, Hill et al. (1993) showed that static strength was not impacted by eastbound travel across five time zones (Hill et al., 1993), whereas Lagarde et al. (2001) reported that static strength was lower by 4% following an eastward travel with a seven time zones shift. Of note, in the former study (Hill et al., 1993), the influence of traveling eastbound on grip strength was evaluated in relatively fit kinesiology majors and the influence of travelling westbound was evaluated in national team football (soccer) athletes, while the latter study (Lagarde et al., 2001) employed volunteers with unspecified fitness/athletic level.

### 5.2 Dynamic strength

Dynamic strength has been shown to be affected by westbound transmeridian travel (Hill et al., 1993; Wright et al., 1983). Wright et al. (1983) reported that travel across five time zones reduced isokinetic shoulder press‐plus‐pulldown strength by 13% at a slow speed and by 9% in fast speed testing, on the first day at the destination.

### 5.3 Power

The impact of an actual westbound travel on sprinting and jumping ability is not certain yet. Chapman et al. (2012) observed that an eastbound flight from Australia to Canada crossing eight time zones reduced jumping ability of skeleton athletes for 11 days postflight. Additionally, Everett et al. (2022) reported meaningful changes in vertical jump velocity and displacement in highly trained rowers who travelled from Canberra, Australia to Milan, Italy crossing nine time zones. Westbound travel (from Sydney, Australia to London, UK) was found to suppress jump height up to 2 days after arrival (Fowler, Duffield, Morrow, et al., 2015). However, another study following the same simulated journey showed no effect on vertical jump or measures of peak power and peak velocity (Fowler, Duffield & Vaile, 2015). In line with this, Fowler, Knez, et al. (2017) reported that both eastbound (from Qatar to Australia) and westbound (from Australia to Qatar) flights had adverse effects on jumping performance. More pronounced effects, however, were shown following eastbound travel. It was also reported that sprinting performance (which we attribute to anaerobic power) was impaired for 3 days following travel, whereas jumping performance (which we attribute to muscle power) was impaired for only 3 days; this supports our contention that the two tests reflect different physiological characteristics, and that these characteristics are affected differently by transmeridian travel.

As noted above in the discussion of aerobic and anaerobic measures, somewhat contradictory results among studies may be explained by differences among studies in (i) travel: direction and duration, during daytime or night times hours, (ii) testing: what was tested (isometric strength, dynamic strength or power) and how, and (iii) timing of the testing: comparison of baseline scores at a particular time of day versus scores at different times of day. Although we have discussed anaerobic power and muscle power separately, we do note that the tests of anaerobic power such as work in 5 s in a Wingate test or performance in a 20‐m sprint, activities of about 3 to 5 s, are certainly impacted by anaerobic energy provision, muscle strength, muscle power and neuromuscular characteristics.

Collectively, a series of studies, but not all, have found that muscle strength and power decline following either eastbound or westbound travel. Performance deterioration is expected to last up to 3 and 4 days after traveling west and east, respectively.

## 6 EFFECTS OF TRANSMERIDIAN TRAVEL ON PERCEPTUAL/PSYCHOPHYSIOLOGICAL VARIABLES

Thus far, different psychometric measures have been used to detect the impact of long‐haul airline travel on the psychological state and the perception of athletes including the Profile of Mood State (POMS) (Hill et al., 1993; O'Connor et al., 1991), the Recovery‐Stress Questionnaire for Athletes (REST‐Q) (Fullagar, Duffield et al., 2016), as well as the self‐reported wellness questionnaire (Buchheit et al., 2016). Regardless of the psychometric measure, most of the above mentioned studies suggest that irrespective of direction, transmeridian travel has adverse effects on athletes’ physical feeling and perception of fatigue. Travelling west in particular, and irrespective of the type of the flight (actual or simulated), has been documented to reduce physical feeling (i.e., participants felt worse compared to pretravel) (Fowler, Duffield & Vaile, 2015), vigour (Fowler, Duffield, Morrow, et al., 2015; Hill et al., 1993) and subjective sleep quality (Reilly et al., 2001), as well as to increase the resting feeling of fatigue (Hill et al., 1993) and the perceived exertion during exercise (Fowler, Duffield & Vaile, 2015; Table 1). By contrast, others have shown unchanged perception (i.e., subjective mental, emotional and physical well‐being; Fullagar et al., 2016), and feelings of fatigue (Fowler, Knez, et al., 2017) or even improved subjective feelings (O'Connor et al., 1991) after actual westbound travel. The latter finding is likely due to the relatively short duration of travel requiring only four time zone shifts. Whatever the case, the negative effects of travelling west on athletes’ perception may dissipate 1–2 days after arrival at the new destination (Hill et al., 1993).

To date, two studies have explored the impact of simulated flights on perceptual and performance aspects of well‐trained participants (Fowler, Duffield, Morrow, et al., 2015, Fowler, Duffield & Vaile, 2015), while other studies have employed real flights (e.g., Bullock et al., 2007; Hill et al., 1993). Furthermore, six studies have examined the impact of traveling east using actual flights on athletes’ perception (Biggins et al., 2022; Buchheit et al., 2016; Fowler, Knez, et al., 2017, Fowler, McCall, et al., 2017, Fowler, Knez, Thornton, et al., 2021; O'Connor et al., 1991). Again, the majority of them indicate that crossing four or more time zones has a deleterious effect on several, but not all, psychometric indices. For example, subjective sleep quality, motivation and general well‐being scores significantly decrease and perceived fatigue increases after long‐haul eastward travel (Buchheit et al., 2016; Fowler, Knez, et al., 2017, Fowler, McCall, et al., 2017, Fowler, Knez, Thornton, et al., 2021), while other indices such as mood and internal training load remain similar to pretravel values. Concerning the time required for perception restoration, two studies have shown that subjective sleep quality and overall well‐being are suppressed for 2–6 days following arrival (Buchheit et al., 2016; Fowler et al., Fowler, McCall, et al., 2017). In line with psychometric responses, psychophysiological measurements such as heart rate variability have been used in one study (Dranitsin, 2008) to detect changes in readiness to train following transmeridian flights. It was observed that, following an eastbound travel with five time zones shift, both supine and standing heart rate variability remained suppressed from the eighth to twelfth day after arrival, thus indicating that readiness to train/compete is probably reduced and may require more than 12 days to be restored. It should be noted that Dranitsin (2008) incorporated a heat acclimatization protocol, which might have affected the results.

## 7 FACTORS THAT MAY DETERMINE THE IMPACT OF RAPID TRANSMERIDIAN TRAVEL ON PERFORMANCE‐RELATED VARIABLES – CONTRIBUTING FACTORS IN THE REAL WORLD, CONFOUNDING FACTORS IN RESEARCH

In the previous sections, we have summarized the reported effects of transmeridian travel on aerobic power, anaerobic power and capacity, muscle strength and power, and various psychophysiological variables. In each case, we mentioned how characteristics of the travel, testing, and timing of the testing, might have affected either the results or the interpretation of the results. In this section we summarize evidence supporting the potential effect of these various characteristics.

### 7.1 Travel

An important feature of travel is the cabin pressurized environment to which individuals are exposed for several hours during transmeridian flights. As it was previously reported, the prolonged mild hypoxic exposure to the cabin pressured environment during transmeridian flights may contribute to the postflight fatigue and jet‐lag related disorders (Coste, Beaumont, Batejat, Van Beers, Charbuy, et al., 2004, Coste, Beaumont, Batejat, Van Beers, Touitou, 2004, 2005; Coste et al., 2009). All travel‐related characteristics including real or simulated conditions (east or westbound, duration, time of day) may induce circadian misalignment. Both mild hypoxic exposure and travel characteristics may desynchronize the biological function, thus contributing to enhanced jet lag symptoms and likely affecting performance.

### 7.2 Testing

Assessment using specific‐to‐sport tests is critical to conclude on performance changes. Most studies reviewed have used YYIRT, which does not offer specific information for most of the individual sports. Another important variable, aerobic power, is usually quantified by ${\dot{V}}_{O_{2}\max}$ or identification of threshold intensities such as the lactate threshold, ventilatory threshold or critical power. And yet, to date, no study has included measurement of any of these variables. It seems that repeated long duration (i.e., 15–30 min) tests are difficult to apply for aerobic power or capacity evaluation when motivation or familiarization may be added as confounding factors. Similarly, isometric strength or short duration power cycling tests may not be relevant to actual competitive performance of elite athletes. In contrast, short duration sprinting or jumping tests may be relevant to team sports. Performance in sprinting and jumping deteriorated, especially following an eastbound travel.

Arguably, one of the most important physiological qualities – for many team sports, sports with 1–5 min rounds (boxing, fencing), any constant power activity of 1–3 min duration – is anaerobic capacity. A wonderful example of the importance of anaerobic capacity is seen in ice‐hockey, where ∼45‐s shifts are designed to get the most production from a player before they face declining anaerobic contribution. And yet, to date, there is only one paper (Hill et al., 1993) that reported a measure of anaerobic capacity after transmeridian travel; that study is over 30 years old and, although it did include separate studies of eastbound and westbound travel, it used non‐athletes, relatively small sample sizes, and a non‐physiological performance‐based measure of anaerobic capacity, the 30‐s Wingate test.

### 7.3 Timing of testing

When one tests at a certain time of day at the destination, it is a different time for the endogenous rhythms, as the body is still transitioning from home town time. The challenge – for the researcher at least – is to determine if reduced performance is caused by jet lag, sleep disruption or circadian rhythmicity. Several studies have addressed the timing of testing, notably as it affects sleep disruption and sleep loss, but also as it affects the persistence of circadian rhythms in the dependent variables. The current evidence suggests that circadian misalignment should be considered in data interpretation after a transmeridian travel.

## 8 PERSPECTIVES

There is no doubt that long‐haul transmeridian travel causes upheaval in most of the psychobiological variables connected to human performance. However, the mechanism of jet lag is far less clear, and we propose that several moderating variables should be considered in future research. We have reported on the literature regarding the possible effects of (i) travel, (ii) testing, and (iii) timing. Here, we shall briefly provide a rationale for including three other potential factors in the discussion of what might mitigate or exacerbate the effects of the impact of rapid transmeridian travel. The three potential factors are (iv) teams, (v) traits, and (vi) tournaments.

### 8.1 Teams

Briefly, different teams might respond differently to transmeridian travel because their performance depends on different factors, which may not be affected to the same degree by travel. For example, it might be expected that a cross‐country team, for which performance is based on aerobic power/endurance, might suffer less than a wrestling team, for which strength, power and anaerobic capacity are very important. This means that research about, or recommendations for, one team may not be pertinent to another. We also offer the possibility that team dynamics might influence susceptibility to travel.

### 8.2 Traits

Our review of the literature suggests that there are insufficient data to address this question. Other characteristics that might influence the response to travel include sex, travel experience, locus of control, chronotype and potentially many others. No study has included men and women in sufficient numbers to compare their responses, and no study has reported travel experience.

Locus of control refers to an individual's perception about the underlying main causes of events in their life. An individual with a strong internal locus of control would believe that they determine their future, whereas an individual with an external locus of control ascribes life experiences to ‘fate’ – que sera, sera. In an early study, we hypothesized that an internal locus of control would ‘protect’ an individual from external challenges, such as sleep loss (or rapid transmeridian travel). It was found that, in 14 participants with an internal locus of control, the total mood disturbance detected by the POMS questionnaire was not significantly elevated (+13), whereas in the 14 participants with an external locus of control, the total mood disturbance detected by the POMS questionnaire was significantly elevated (+33) (Hill et al., 1996). Given the link between changes in mood state and changes in various physiological measures, we hypothesize that locus of control may be a factor in the severity of jet lag symptomology. However, our review of the literature finds no evidence that this possibility has been explored.

Chronotype describes a general pattern among various circadian rhythms of an individual. It is an individual's characteristic preference toward morningness and eveningness and is usually evaluated using self‐assessment questionnaires, among them the classic Horne and Östberg (1976) morningness–eveningness questionnaire and the shorter version of Vitale & Weydahl (2017). Previous results from our lab (Hill & Chtourou, 2020; Hill & Smith, 1991; Marth et al., 1998) reported that anaerobic capacity (work in a 30‐s Wingate test) improved by 0.8 kJ (5%) from 08:00 to 20:00 in seven morning types and by twice as much, 1.6 kJ (10%), in seven evening types. Interestingly, total mood disturbance quantified by the POMS questionnaires, was lower in the morning (94 ± 9) than in the afternoon (100 ± 10) and evening (120 ± 9) for the morning types whereas the opposite pattern was displayed by the evening types; total mood disturbance was higher in the morning (117 ± 8) than in the afternoon (109 ± 4) or evening (106 ± 10) for the evening types. Moreover, for the morning types, the morning–afternoon differences in performance were related to differences in feelings of vigour, and the afternoon–evening differences in performance were related to differences in feelings of fatigue; in contrast, for the evening types, the morning–afternoon differences in performance were related to differences in feelings of fatigue, and the afternoon–evening differences in performance were related to differences in feelings of vigour.

In this discussion of chronotype, we have introduced the relationship between mood state and performance. Traditionally, if an intervention causes a worsening of mood but no change in performance, we would conclude that mood state and performance are not related. This was the case in a recent study (Hill & Chtourou, 2022). So, while the group's performance was not affected, certain individuals were, and individual changes in mood state following sleep deprivation were correlated with individual changes in performance (Hill & Chtourou, 2022). Interestingly, in that study, in the first 24 h following a sleepless night, performance decrements were related to increased feelings of fatigue, whereas during the second day after the sleepless night, performance decrements were related to decreased feelings of vigour. Thus, there is a complicated relationship between mood state disruption and performance, which likely is also evident in the responses to transmeridian travel. Researchers must be careful to investigate individuals’ responses and not just mean responses.

Altogether, the findings from our lab that demonstrate interactions between circadian rhythms, disruption of circadian rhythms, chronotype, mood state and performance measures of anaerobic capacity have led us to hypothesize that chronotype may influence responses to travel. Evidence that evening types might have greater susceptibility to eastbound (phase‐shift‐advance) travel (they are trying to sleep when their bodies are primed for physical activity) (Kori et al., 2017) supports our hypothesis, although studies of ‘social jet lag’ or ‘Monday morning jet lag’ that occurs after later‐than‐usual bedtimes during the weekend have been reported to be worse in morning types (Bottary et al., 2020), although only two of their 26 participants were actually morning types. In addition, we should recommend that future studies should be conducted planning more field‐based experiments, including performance‐related physiological variables.

### 8.3 Tournaments

Athletes travelling for important sport events (e.g., Olympics and World championships) are at the highest level of their sport, and they are able to focus on upcoming tournaments despite many distractions. We hypothesize that the purpose of travel – to a tournament or from a tournament – might impact the severity of jet lag symptoms, especially with respect to physical performance. While the intent of travel may not influence the physiological effects of circadian desynchronization on performance, it may well influence the psychological (and psychophysiological) responses. We propose that, for athletes travelling to a tournament with a focus on the tournament, ‘jet lag’ or the inconveniences of travel are simply a distraction, which they can ignore. Athletes may not experience the mood disturbance typically associated with travel because they are focused on the purpose of the upcoming tournament. This would have two implications: first, athletes travelling to a tournament will not report the mood state disruption that is considered a hallmark of jet lag and, if there is any kind of causal relationship between mood and performance (Hill and Smith, 1991; Hill & Chtourou, 2022) then the impact of travel on performance will be mitigated. However, we caution, while there is little published information to support our suggestions that teams or tournaments may play a role in the severity of jet lag, there is some evidence to support our assertion that there may be a link with traits and the severity of jet lag.

## 9 CONCLUSIONS

This critical appraisal of the extant literature reveals that anaerobic aspects of performance (anaerobic power, muscle power) and strength are negatively impacted by long‐haul transmeridian travel. The existing evidence concerning the effects of transmeridian travel on endurance performance is equivocal. Regarding flight direction, it seems either westbound or eastbound travel can reduce exercise performance, but the negative effects would be more pronounced after eastbound travel. In any case, performance is reduced and it may need more than 3 days to be restored. It is clear that the rule of thumb that humans need 0.5 and 1 day per time zone crossed west and east, respectively, to be fully aligned with the new light–dark cycle does not apply to all athletes. Many factors may influence the time needed for recovery after rapid transmeridian travel, and much more research is needed to determine their role. These factors include the following: (i) travel: direction of travel (in general it appears that eastbound travel is more disruptive than westbound travel, but the duration of the effects is less clear) and number of time zones (in general it appears that the greater the number of time zones crossed, the greater the effect, but, again, the duration of the effects is less clear); (ii) testing: specifically, there are tests that are relevant to team sports but not to individual athletes; it is likely that travel (like sleep deprivation) has different effects on different biological systems; and (iii) timing: both the timing of travel and its effect on sleep and the timing of testing, which is confounded by underlying circadian rhythmicity. Other contributing factors may include (iv) teams, (v) traits, and (vi) tournaments. However, it is not yet possible to conclude that there is any particular amount of time needed for athletes to recover from transmeridian travel.

## AUTHOR CONTRIBUTIONS

All authors wrote, drafted, and critically revised the paper throughout its inception. All authors have read and approved the final version of the manuscript. All authors agree to be accountable for all aspects of the work to ensure that questions related to the accuracy or integrity of any part of the work are appropriately investigated and resolved. All persons designated as authors qualify for authorship, and all those who qualify for authorship are listed.

## CONFLICT OF INTEREST

None declared.
